# Disease Control Priorities Third Edition: Time to Put a Theory of Change Into Practice: Comment on "Disease Control Priorities Third Edition Is Published: A Theory of Change Is Needed for Translating Evidence to Health Policy"

**DOI:** 10.15171/ijhpm.2018.115

**Published:** 2018-11-28

**Authors:** Wanrudee Isaranuwatchai, Ryan Li, Amanda Glassman, Yot Teerawattananon, Anthony J. Culyer, Kalipso Chalkidou

**Affiliations:** ^1^Health Intervention and Technology Assessment Program, Ministry of Public Health, Nonthaburi, Thailand.; ^2^Institute of Health Policy, Management and Evaluation, University of Toronto, Toronto, ON, Canada.; ^3^Centre for Excellence in Economic Analysis Research, St. Michael’s Hospital, Toronto, ON, Canada.; ^4^School of Public Health, Imperial College London, London, UK.; ^5^Center for Global Development, London, UK.; ^6^Department of Economics and Related Studies and Centre for Health Economics, University of York, York, UK.

**Keywords:** Priority Setting, Theory of Change, Disease Control Priorities, Health Technology Assessment, Economic Evidence

## Abstract

The Disease Control Priorities program (DCP) has pioneered the use of economic evidence in health. The theory of change (ToC) put forward by Norheim is a further welcome and necessary step towards translating DCP evidence into better priority setting in low- and middle-income countries (LMICs). We also agree that institutionalising evidence for informed priority-setting processes is crucial. Unfortunately, there have been missed opportunities for the DCP program to challenge ill-judged global norms about opportunity costs and too little respect has been shown for the wider set of local circumstances that may enable, or disable, the productive application of the DCP evidence base. We suggest that the best way forward for the global health community is a new platform that integrates the many existing development initiatives and that is driven by countries’ asks.

## Introduction

The Disease Control Priorities program (DCP) generated influential volumes of evidence for informing global health spending priorities and catalysed an international culture shift in the use of economic evidence for health. There is, however, still far to go before countries actually have the capacities to set their own priorities for achieving and sustaining universal health coverage (UHC).

Since DCP1, the project’s main audience has been global funders and advocates. The next challenge is how best to engage governments. It has been proven repeatedly that making priority setting evidence available is not enough alone to bring about policy change.^1^ Norheim proposes a theory of change (ToC) for “better priority-setting.”^2^ The ToC sets out the causal steps and assumptions that would enable the achievement of short-term DCP outcomes as well as intermediate- and long-term country outcomes. The steps include building capacity, institutionalising priority-setting and, the ultimate goal: resource allocation informed by epidemiological and economic evidence. Such a ToC would be a natural next step for DCP, whose sole mission to date has been generating global evidence, but evidence that has not been tailored to the needs of specific countries.

Norheim underlines the importance of country context. He cautions that not all contextual factors can be influenced by programs like DCP. However, DCP, together with like motivated initiatives such as the International Decision Support Initiative (iDSI) and the global health community, may be able to deliver the ultimate goal. *Together*, these initiatives can go far to shape contexts and build supportive environments for low- and middle-income countries (LMICs) aiming at UHC.

## Taking Pride in Cost-Effectiveness

DCP1’s greatest achievement was that it got the global development community to recognise the importance of maximising the impact of healthcare resources through cost-effectiveness analysis. It catalysed an international culture shift towards the use of economic evidence in health resource allocation. As Norheim^2^ points out, this shift has been embraced by many countries, the World Health Organization (WHO) and major donors, including the Bill and Melinda Gates Foundation. The Foundation remains one of the world’s biggest largest external funders for global health and exerts huge influence both directly and indirectly via conduits such as the Global Fund and Gavi.^3^

Norheim draws attention to criticisms of earlier versions of DCP, notably the use of cost-effectiveness as a key criterion for decision-making to the exclusion of affordability, ethics, equity, legitimacy and human rights. These criteria are, however, far from being inherently incompatible with cost-effectiveness – but they need integrating into a more comprehensive method of appraisal and implemented through institutions that understand the importance of each. Cost-effectiveness is already a form of applied ethics (a species of utilitarianism) but needs further supplementation^4-7^ and appropriately designed institutions and decision-making procedures. It is unquestionably true that “health policy needs to go beyond cost-effectiveness”^2^ but this ought not to be interpreted in a negative way. Cost-effectiveness needs to be supplemented not supplanted: “where economic evidence is down-valued or even deprioritised over other socially acceptable considerations…risks throwing the baby out with the bathwater.”^8^ The world needs more, not less, cost-effectiveness analysis^8^ – so long as it respects each country’s values and circumstances.

As Norheim notes, DCP3 centred on advancing UHC with a focus on economic feasibility by selecting the “highest priority package that could be implemented first and at less cost.” Yet, the cost of the highest priority package was typically still larger than the average public spend per capita on health in low-income countries.^9^ One needs to ask: economic feasibility for whom? and “feasibility from what perspective?” These are the first questions any useful economic analysis should address. The global health community has paid insufficient attention to these issues.^10^ It is as though differences in history, culture, social values, priorities for other sectors, political feasibility, and available budgets were not significant differentiating factors among nations. This may partially explain why the real-world uptake of DCP and other economic evidence in resource allocation decisions has been disappointing. It often simply did not fit.

## Opportunity Costs and Missed Opportunities

Despite the WHO’s and the World Bank’s cost-effectiveness advocacy, DCP has not used its significant global influence to challenge questionable global norms. For instance, Millennium Development Goals (MDGs) targets are disconnected from national budgets, as are WHO Standard Treatment Guidelines^11^ and Essential Medicines Lists.^12^ Until recently,^13^ WHO CHOICE was associated with the 1-3x gross domestic product (GDP) guidance for willingness-to-pay thresholds, which generally underestimate actual opportunity costs of health interventions in LMICs.^14^ The ill effects can be lasting. WHO has not replaced its threshold recommendations with any new practical guidance, so countries struggle to apply health technology assessment (HTA) while many researchers and public servants mistakenly continue to use 1-3x GDP in the absence of anything better. Such guidance can do more harm than good for LMICs already struggling to achieve UHC within constrained budgets.^15^

If policy-makers are to look to DCP for guidance, a good start for DCP would be to acknowledge the importance of the constraints and opportunity costs that underpin thresholds and budget impacts. Much greater emphasis also needs to be placed on data and the interpretation of data from outside the country. There is now substantive work on the transferability of evidence between contexts. Strengthening the robustness and local applicability of DCP evidence would increase its global credibility and simultaneously make DCP knowledge more readily interpretable in individual country settings.^1,16^

## Putting a Theory of Change Into Practice

DCP-style evidence has not been generated in response to specific local needs. Nonetheless, there have been successful attempts to adapt and apply DCP locally to facilitate change. In the Philippines, global DCP evidence filled a local gap and informed sound policy decisions serving “as a credible, temporary solution…while countries continue their efforts in developing local capacity to generate data.”^17^ Lists of priority interventions were “a necessary first step to start a more formal priority setting process” in the Philippines intended eventually to be housed in an independent HTA agency,^17^ which will advise the Ministry of Health on service expansions.

Having country-specific ToCs for translating economic evidence into policy decisions and institutionalising this process would greatly enhance the usefulness of extended cost-effectiveness analysis and would be a step towards the local targeting of evidence generation. Norheim’s proposed DCP ToC bears similarities to the iDSI ToC (Figure).^18^ We have successfully tried-and-tested the iDSI ToC for building countries’ priority-setting capacities and establish institutional mechanisms which in turn result in evidence-informed, cost-effective and equitable decisions.^19^

**Figure:**
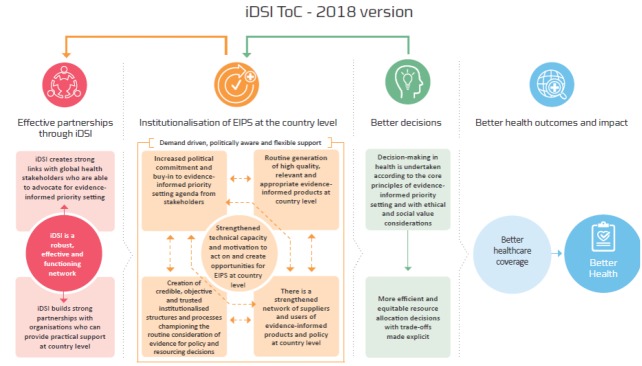
The International Decision Support Initiative’s (iDSI’s) Theory of Change (ToC).

Norheim leaves unanswered a big question: whose responsibility is it to operationalise DCP’s ToC? Does DCP see itself evolving as an evidence generating initiative, a force for capacity-building, a technical assistance initiative, or all three? Norheim is right that priority-setting is ultimately the responsibility of each country (or consortia of like-minded countries), yet there is a clear unmet need to support countries’ capacity-building efforts to conduct and interpret research^16^ and to institutionalise the capacity for such things. Help, for the time being, will need to come from outside. DCP ought to be a part of that help but it cannot provide it alone.

## Going Further as a Global Community

The global health community should move beyond a piecemeal, project-by-project approach to research, beyond advocacy and exhortation, and beyond knowledge sharing. It must not continue powerless to shape the contextual factors that Norheim considers critical for sustainable systems of effective resource allocations that generate best value for money in health.

We need a global community of practice that diffuses global knowledge into practice through country-led and country-owned policy mechanisms. This community could include DCP, iDSI, Global Burden of Disease, Tufts’ Cost-Effective Analysis registry, HIV/TB/malaria modelling consortia, Global Health Costing Consortium, Joint Learning Network, and capacity building platforms (eg, Strategic Purchasing African Resource Center), and others.

Such a community would be a truly grand consortium for transitioning LMICs^20^ through coordinated, national-level reforms delivering comprehensive and affordable UHC packages. The aspiration gap is enormous^21^ but the demand is real. DCP, with iDSI and other like-minded initiatives and funders, can together articulate that joined-up offer. We suggest a new platform, in which multiple initiatives are merged and driven by the needs articulated by individual countries, is the way to do it.

What sort of platform might this be? We would welcome ideas on how this might be achieved with minimal interference with the independence of but in such a fashion as to help LMICs develop strategies for creating supportive political and professional environments for the application of cost-effective evidence in decision about public insured benefits. This should be supplemented where appropriate by evidence on other criteria (such as population coverage, equity and financial protection, and including Norheim’s criteria), and further supplemented by the establishment of agencies or specialised in-country groups to provide and evaluate such evidence and by training programs both for building capacity in the technical skills needed and for the non-technical partners in decision making processes. Not least, work on informing the wider public of health service users and taxpayers would further complement these efforts.

That these intentions would be better for being coordinated in some fashion seems to us to be self-evident. Would it be worth the effort – and whose effort it ought to be? On this, we would welcome a debate.

## Ethical issues

Not applicable.

## Competing interests

Authors declare that they no competing interests.

## Authors’ contribution

All authors contributed to the conceptualization and writing of this paper.

## Authors’ affiliations

^1^Health Intervention and Technology Assessment Program, Ministry of Public Health, Nonthaburi, Thailand. ^2^Institute of Health Policy, Management and Evaluation, University of Toronto, Toronto, ON, Canada. ^3^Centre for Excellence in Economic Analysis Research, St. Michael’s Hospital, Toronto, ON, Canada. ^4^School of Public Health, Imperial College London, London, UK. ^5^Center for Global Development, London, UK. ^6^Department of Economics and Related Studies and Centre for Health Economics, University of York, York, UK.
